# Re-creation of the Inframammary Fold, Breast Mound, and Projection by the Latissimus Dorsi Musculocutaneous Flap in Burned Breasts

**Published:** 2011-02-24

**Authors:** Seyed Mehdi Mousavizadeh, Sadrollah Motamed, Seyed Nejat Hosseini, Parvin Yavari

**Affiliations:** ^a^Plastic and Reconstructive Surgery Department, 15 Khordad Hospital, Shahid Beheshti University of Medical Sciences, Tehran, Iran; ^b^Zanjan University of Medical Sciences, Zanjan, Iran; ^c^Digestive Disease Research Center, Shariati Hospital, Shahid Beheshti University of Medical Sciences, Tehran, Iran

## Abstract

**Objective:** Some of the most difficult problems to solve in a postburn breast are the correction of the breast mound deficiency, contour, and projection deformity, which are often associated with an anterior trunk scar. The aim of this study was to describe our experiences of postburn breast reconstruction by the island latissimus dorsi musculocutaneous flap (LDMCF). **Method:** Operative procedures were planned after measuring the volume, dimensions, sternal notch-to-nipple distance, deviation, asymmetry, contour, and projection. Scar contracture release was carried out and complete muscle elevation was performed in all patients. The size of the skin paddle depended on the envelope deficiency. Afterward, LDMCF was transferred by a subcutaneous tunneling, and the muscle was sutured in retroglandular, inferomedial, and inferolateral ways to shape the inframammary fold (IMF) contour, breast mound, and projection. The skin of the flap was trimmed to match the envelope deficiency. **Result:** A total of 9 burned patients (11 breasts), who had burnt anterior trunks due to scalds and flame, entered the study. They were reconstructed by LDMCF. The patients achieved breast contour (re-creating the IMF), projection, and breast mound increase. The means of breast mound and projection increase were about 140 mL and 2.5 cm, respectively. **Conclusions:** This study demonstrates that the method used for reconstructing the burned breast depends on the patient's clinical presentation. For the patients with anterior trunk scar who have breast mound deficiency, IMF, and projection deformity, LDMCF is one of the best options of reconstruction.

Chest wall burns in women can create a wide variety of problems such as scars, damaging the nipple areolar complex and also the breast contour and volume. Also, aesthetic considerations play an important role in how a breast is reconstructed. The conventional treatment for breast contour or inframammary fold (IMF) deformities entails contracture release followed by incision or excision of the restricting burned scar and insertion of a thick split thickness skin graft.^1^ Other techniques may include local skin flaps, Z plasty, tissue expansion and mammary prostheses and musculocutaneous flaps.^2^^-^5 Naturally, in addition to the sense of well-being, the patient with a breast deformity who presents herself to a reconstructive surgeon is looking for the opportunity to restore her emotional health too. Hence, the important challenge is to create an aesthetically pleasing breast. In this respect, in order to achieve the proper reconstructive result, there are a myriad of surgical procedures for modifying and reconstructing breast deformities.^2^

Some of the most difficult problems to solve in a postburn breast are the correction of the breast mound deficiency, contour, and projection deformity, which are often associated with an anterior trunk scar. The burn pattern, breasts envelope and the patient's expectations may affect the option that is chosen for reconstruction. The aim of this study was to describe our experience of postburn breast reconstruction by the island latissimus dorsi musculocutaneous flap (LDMCF) in young women who had IMF, breast mound deficiency, and projection deformity.

## PATIENTS AND METHODS

**Figure 1:**
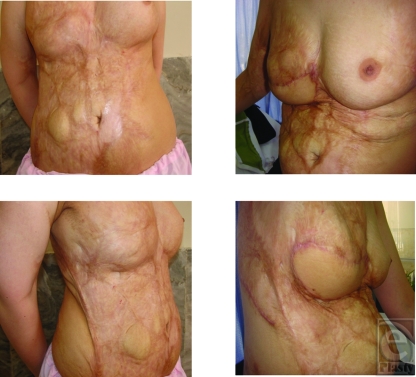
A patient with thick scar, inframammary fold, breast mound, and projection deficiencies before and after surgery.

**Figure 2:**
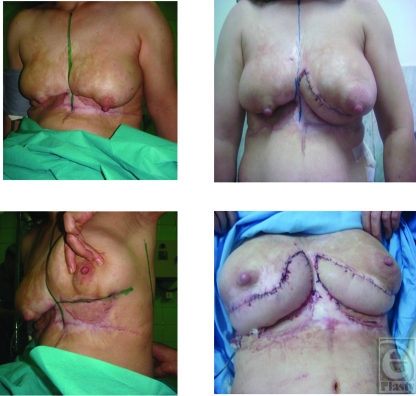
A second patient with thick scar, inframammary fold, breast mound, and projection deficiencies before and after surgery.

Over a 3-year period from October 2005 to February 2008, many patients with breast deformity and anterior trunk scars were referred to our center for breast reconstruction. From among them, 44 patients who were referred to our center had abnormal breast development due to restrictive skin envelope, breast tissue damage, and lack of inframammary crease. All their deformities were due to prepubertal burns. Operative procedures were planned after measuring the volume, dimensions, sternal notch-to-nipple distance, deviation, asymmetry, contour, and projection. Only 9 patients with anterior trunk scars, including those with moderate breast mound deficiency, contour (IMF or inferior, medial, and lateral contour), and projection deformities, were chosen to receive LDMCF (Figs 1 and 2).

Patients with posterior trunk burns or with no burns on the lower abdomen and those who did not consent to any major surgery were excluded from the study. However, 3 patients with no burns on their lower abdomen who were not willing to accept the transverse rectus abdominis myocutaneous (TRAM) flap because of pregnancy, work issues, or hernia were also selected to receive LDMCF. Also, it should be mentioned that previously, skin grafts for IMF reconstruction and Z-plasty had been performed on 4 of the patients (ie, 6 breasts) in some other centers. However, the patients were not satisfied with the results, because they suffered from a poor body image and consequently bad emotional health (Table 1). In spite of their high motivation, they were unable to find partners for marriage.

Complete information about the procedure was presented to all patients in advance (written and oral). In addition, informed consent was taken from all the patients prior to their enrollment in the study. They were notified about the multistage process of the reconstruction and became aware of the possibility of late scar formation. On the whole, 11 LDMCFs were undertaken in 9 patients without any systemic disease. Lastly, the average follow-up period was 14 months (ranging from 6 to 34 months). This study was approved by the ethics committee of Shahid Beheshti University of Medical Sciences.

## SURGICAL TECHNIQUE

**Figure 3:**
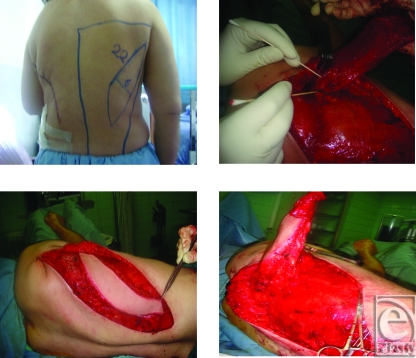
The axis of LD and the skin paddle design.

Before the operation, the contracted breast scar area that was planned to be released was marked with the patient in an upright position. The primary LDMCF design was also performed in this position (Fig 3). Afterward, by putting the patient in the supine position and releasing the scar contracture under general anesthesia at the inferior, medial, and lateral contours, the glandular tissues were partially elevated from the chest wall. Then the breast mound and envelope deficiency was determined. Breast release was performed from the fourth to the sixth rib, and approximately 2 cm from the sternal edge to the anterior axillary line. Next, the patient was turned to the lateral decubitus position so that the size of the skin paddle, fat, and latissimus dorsi (LD) muscle was marked. It should be mentioned that the exact design of the skin pattern was determined intraoperatively. The procedure required all the available excess of the posterior trunk skin and fat when IMF and breast mound reconstruction was supposed to be performed. Afterward, the axis of LD flap was determined and the LD muscle was released from the other tissues of the posterior trunk (Fig 3).

When the anterior border of the LD muscle was dissected, the skin and the muscle were slightly retracted to confirm vascular distribution. The pedicle's artery and the flap's vein were identified. However, the neurovascular pedicle was not dissected. The humerus insertion was cut from the LD muscle. Then the LD muscle with its overlying skin paddle was lifted from the posterior trunk to the inferior border of the breast by subcutaneous tunneling like the island flap.

Complete LD muscle elevation was performed in all patients but the size of skin paddle depended on the individual's skin deficiency and their differences. Minimum and maximum widths of skin paddle were 6 and 8 cm, respectively. That is why the volume of the flap was calculated according to the formula: volume = thickness × length × width × 0/81.^6^ The donor site was primarily sutured and the suction drains were inserted. Then the patient was turned to the semilateral position or nearly supine position for insetting the flap. The skin of the flap was trimmed to match the envelope deficiency. The breast tissue was subsequently repositioned to achieve a normal shape and position. Also each breast was compared to its contralateral side to achieve symmetry. Assessing and determining the extent of the contracture and breast mound deficiency to compare to the other breast was carried out previously in the upright and supine position. The LD muscle was sutured in the retroglandular, inferomedial, and inferolateral aspects to shape the IMF contour, breast mound, and projection. A drain was also inserted in the retroglandular space of the breast. Finally, the wound was closed with absorbable sutures.

## RESULT

A total of 9 patients (ie, 11 breasts) were chosen for this study. Seven patients had burns on their anterior trunk due to scalds (ie, 8 breasts) and 2 patients had that due to flame (ie, 3 breasts). They had 15% to 46% TBSA and deep second- and third-degree burns, which were not initially treated by excision and grafting (Table 1). Six of these 9 patients had been massively burnt over the anterior chest and abdomen and the rest on the anterior chest only (these 3 patients would not accept a TRAM flap for breast reconstruction). All patients had moderate breast mound deficiency, IMF (inferior, medial, and lateral contour), and projection deformities. The mean time from when they were burned primarily up to their first referral to us was 2 years. The mean age of these 9 patients at the time of first referral was 21. All patients were single. The most common burned areas were the anterior trunk, right breast, neck, right arm, and face. The means of breast mound and projection increase were about 140 mL and 2.5 cm, respectively. Complete muscle elevation and transfer was performed for all patients but the size of skin paddle depended on the amount of skin deficiency (Table 1).

In this study, 11 breasts were reconstructed by LDMCF. After the operation, they had achieved breast contour (reshaped IMF), projection, and breast mound increase. Also, each patient achieved a more pleasing aesthetic result, and emotional health was improved (Figs 1 and 2). The results were satisfactory in all cases, according to the volume measurements and projections. In this respect, we explained 3 levels of patient satisfaction: fair, good, and excellent (based on projection and breast mound increase compared to the preoperative condition and the contralateral breast), which are shown as + +, + + +, and + + + +. The demographic data and all other information are included in Table 1.

## DISCUSSION

The present study supports the hypothesis that using LDMCF is one of the best options for moderate breast mound deficiency and IMF reconstruction. Generally, lack of enough education and low socioeconomic status are considered to be associated with a high incidence of thermal injury.^7^ In the toddler group, scalds are the most frequent cause for referral to burn units. However, flame burns occur more often in older children and are more likely to produce a full-thickness wound.^8^ Both scalds and flame burns often involve the chest. Patients with chest burns require reconstructive surgery after the onset of the breast development. Problems encountered include breast asymmetry, distortion of the breast's shape, and unpleasant skin texture.^9^ In one study, all patients underwent skin grafting, which required reconstructive surgery after the onset of the breast development.^10^ Scarred and distorted breasts after burns can have a significant psychological burden on adolescent girls and young women.^11^

The cause, location, and burn age of our study were similar to those of others. However, in our case, the patients had not received reconstructive surgery in the acute phases. In addition, our patients were in need of breast reconstructive surgery and were referred to us after some years of breast development.

To our knowledge, as far as the literature shows, the most common procedure performed for postburn breast reconstruction has been the release with skin grafting. Other less common methods are cutaneous flaps, Z plasty, musculocutaneous flaps, and skin expansion with implants.^2^^-^5 Whenever the breast mound deficiency had been severe, flap and implant were required for reconstruction.^12^

Restoring the symmetry of the breast can be achieved by reduction mammoplasty or mastopexy of the contralateral breast. In this case, tissue expanders must be used and reverse abdominoplasty be performed, thus re-creating the IMF.^13^ Expansion and implant are prone to complications such as exposure of injection port, infections, spontaneous deflation, exposure of the implants, and capsule formation.^14^,^15^ Some authors even found chest wall deformities as a complication after maximal tissue expansion for breast reconstruction.^16^ Finally, the posterior-lateral expanded flap had been an alternative option for breast envelope creation and IMF reconstruction, but this flap cannot correct the breast mound deficiency.

In our study, the breast mound, IMF, and projection correction were very important for the patients. That is why cutaneous flaps, expansion plus implant, and the TRAM flap were not selected for them. To be more precise, it should be mentioned that tissue expanders and reverse abdominoplasty and TRAM flap were not a good option for some of them (ie, 6 patients) because they had chest scars and abdominal burns. The rest would not accept a TRAM flap because of their susceptibility to scar formation, pregnancy, and hernia formation. In this respect, Bishop has reported that where breast mound loss has occurred, the deficient tissue should be best supplied with a LD flap in a single operation, resulting in a better aesthetic result and minimal hospital stay.^17^

Latissimus dorsi musculocutaneous flap is a well-known flap, initially used in breast reconstruction in association with an implant.^18^ Previous studies have shown that patients have fewer complaints after the LD muscle transfer due to less functional loss.^19^,^20^ Of course, there are other potential options like the superior gluteal artery perforator free flap and deep inferior epigastic perforator, but these techniques are very much dependent on the surgeon's experience.

In this study, LDMCF was used for reconstructing the burned breasts. Although most of the breasts had acceptable volume, mound deficiency, IMF, and projection deformity existed in all patients. In addition, the bilateral LDMCF achieved an acceptable symmetry, and the patients were satisfied with it. However, some of the patients were in need of some other procedures, including nipple areolar complex correction, reduction mammoplasty, or mastopexy. The important point of this study is that before conducting any operations, the breast mound, contour, and projection deformity must be corrected.

## CONCLUSION

This study shows that the methods used for reconstructing a burned breast depends on the patients' clinical presentation. When a patient is referred with such a problem, first of all she must completely be examined. Then the breast mound deficiencies, distortion, counter deformity, scar pattern, and the patient's expectations must be accounted for. In this respect, for patients with anterior trunk scar who have breast mound deficiency, IMF, and projection deformity, LDMCF is one of the best choices of reconstruction.

Finally, the study can be summarized as follows: (1) breast releasing was performed from the fourth to the sixth rib and in retroglandular plane; (2) complete LD muscle elevation was performed in all patients, but the size of skin paddle depended on their envelope deficiency; (3) the neurovascular pedicle was not dissected, and LDMCF was transferred by subcutaneous tunneling; (4) the LD muscle was sutured in retroglandular, inferomedial, and inferolateral aspects to shape the IMF contour, breast mound, and projection; and (5) the skin of the flap was trimmed to match the envelope deficiency.

## Acknowledgments

This study was supported by a grant from the deputy for research of Shahid Beheshti University of Medical Sciences. The authors thank all the staff of the burns and the clinic unit of 15 Khordad Hospital for their sincere cooperation. They also thank Seyed Muhammed Hussein Mousavinasab for his sincere efforts in revising and editing this work.

## Figures and Tables

**Table 1:** Demographic information of the patients who have undergone LDMCF*

N/R or L	Cause	Burned age/Referred Age	Prereferral Surgery	Extension Lesion?	Projection Gradient (cm)	LDMCF Volume (mL)	Flap Complication	Result
1 R	Scald	2, 21	…	BMD, IMF, PD, UST	2.5	160	Distal flap necrosis (2 cm)	+ +
1 L	Scald	2, 21	…	BMD, IMF, PD	2.5	160	No	+ + +
2 R	Scald	2, 19	split skin grafts, IMF	BMD, IMF, PD, UST, loss of NAC, asymmetry	2	170	No	+ + +
3 R	Scald	3, 17	…	BMD, IMF, PD, UST, RUQC, asymmetry	2.5	120	No	+ + +
4 R	Scald	2, 26	Z-plasty on RUQC	BMD, IMF, PD, UST, asymmetry	3	130	No	+ + + +
5 R	Scald	5, 20	…	BMD, IMF, PD, UST, loss of areola, asymmetry	3	130	Tittle (1cm)	
Distal necrosis	+ + ++							
6 L	Scald	1, 24	Z-plasty on RUQC	BMD, IMF, PD, UST	3	150	Left side partial margin necrosis	+ ++
7 L	Scald	1, 22	…	BMD, IMF, PD, UST, Asymmetry	2.5	125	No	+ + +
8 R	Flam	13, 28	split skin grafts, IMF	BMD, IMF, PD, UST	2	140	No	+ + +
8 L	Flam	13, 28	split skin grafts, IMF	BMD, IMF, PD, UST, RUQC	2	140	Tittle (1cm)	
Distal necrosis	+++							
9 R	Flam	11, 26	…	BMD, IMF, PD, loss of NAC, asymmetry	3	130		++++

*IMF indicates inframammary fold deformity; LDMCF, latissimus dorsi musculocutaneous flap; LT, left; BMD, breast mound deficiency; NAC, nipple areolar complex; PD, projection deformity; RT, Right; UST, unpleasant skin texture; RUQC, right upper quadrant contracture.
